# Persistent Hiccups: An Unusual Presentation of Aspiration Pneumonia in the Elderly

**DOI:** 10.7759/cureus.19514

**Published:** 2021-11-12

**Authors:** Lance De Barry, Narika Singh, Triston De Barry

**Affiliations:** 1 Obstetrics and Gynaecology, South West Regional Health Authority, San Fernando, TTO; 2 Internal Medicine, South West Regional Health Authority, San Fernando, TTO; 3 Internal Medicine, The University of West Indies, San Fernando, TTO

**Keywords:** aspiration, pulmonology, chlorpromazine, aspiration pneumonia, persistent hiccups

## Abstract

Persistent hiccups manifesting as the sole symptom of aspiration pneumonia is a rare occurrence. Approximately 10 cases have been reported in the last 15 years. Hiccups are defined as persistent if it occurs beyond 48 hours and intractable if it occurs continuously for one month. We highlight a case of an elderly man diagnosed with a subacute ischemic infarct of the right occipital lobe with a preserved gag reflex and swallow reflex. The patient’s persistent hiccups began eight hours after an emetic episode. Typical signs of pneumonia were absent. Chest x-ray revealed bilateral lower lobe pulmonary infiltrates and he was treated aggressively with intravenous antibiotics and chlorpromazine. He made a full recovery and was discharged four days later.

## Introduction

Aspiration pneumonia is a serious cause of morbidity in the elderly [1]. It is defined as an infectious pulmonary process that occurs when aspirated oropharyngeal secretions or gastric contents enter the lower respiratory tract [2]. Common risk factors for aspiration pneumonia include stroke, alcohol use, seizures, gastroesophageal reflux disease and dementia, among others [1].

Patients typically present with cough, dyspnea, fever, hypoxia, reduced breath sounds and radiological evidence of pulmonary infiltrates [3]. Rarely, patients present with persistent hiccups as the sole symptom of aspiration pneumonia and when it does occur, the inferior lobe of the right lung is typically involved [4,5]. Persistent or intractable hiccups always represent an underlying pathology. Investigations include chest radiography and an evaluation of inflammatory markers. Treatment is aimed at symptomatic relief and the underlying etiology [6].

Herein, we describe a case of aspiration pneumonia presenting as persistent hiccups in an elderly male, recently diagnosed with a subacute ischemic infarct of the right medial occipital lobe. Chest radiograph confirmed bilateral lower lobe pulmonary infiltrates and the patient was treated with antibiotics and a low-dose chlorpromazine infusion. He was subsequently discharged four days later after an uneventful recovery.

## Case presentation

A 73-year-old male presented to the emergency department with persistent hiccups over the past five days. The patient is a known diabetic and hypertensive for 15 years and has rate-controlled atrial fibrillation, managed with beta-blockers and warfarin. Two weeks ago, he suffered a subacute ischemic infarct of the right medial occipital lobe, which was confirmed on a computed tomography (CT) scan of the brain. The patient had both a preserved gag and swallow reflex. The family reported one bout of post-prandial emesis while upright five days earlier, after which the hiccups began eight hours later. The patient had no other symptoms, and the family denies him experiencing fever, chills, dyspnea, cough, malaise, and confusion. 

**Figure 1:**
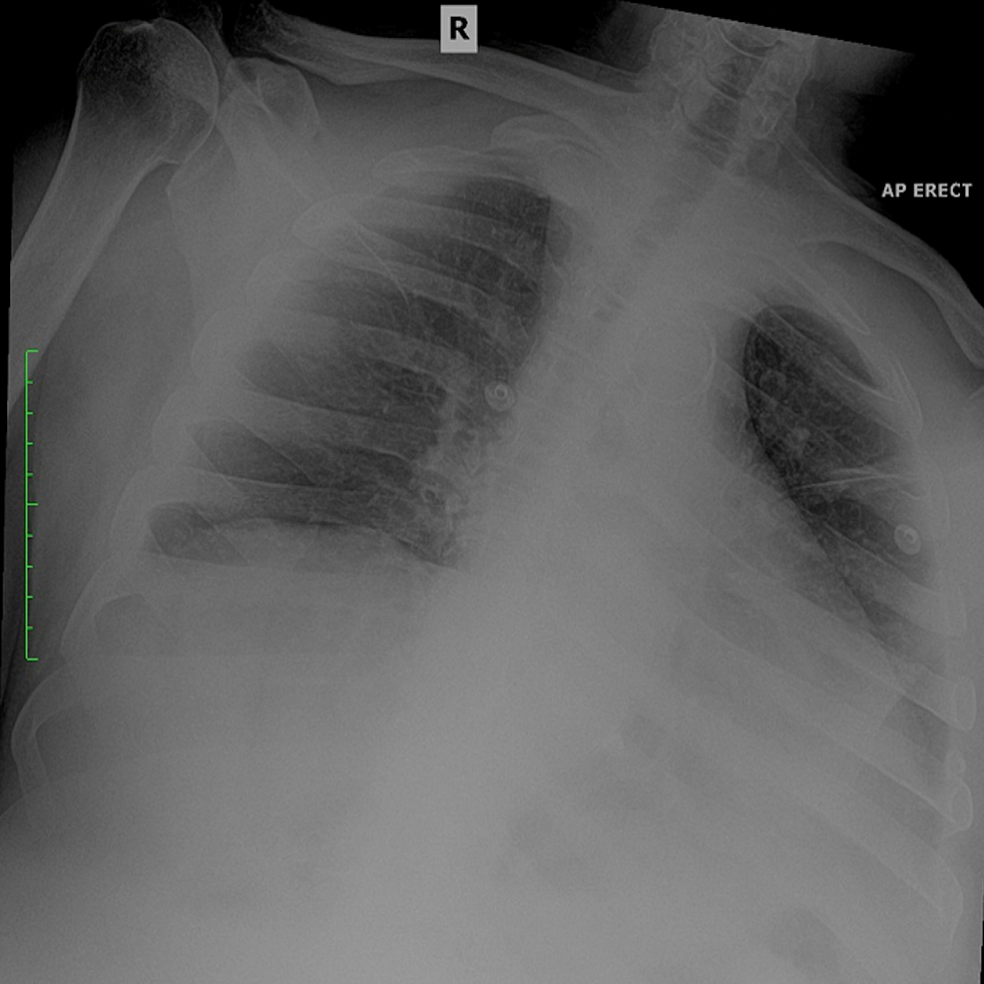
Chest radiograph on admission demonstrating bilateral basal lung infiltrates.

On clinical examination, there were decreased breath sounds and crackles noted in the basal segments of the right lower lobe. The patient had a low-grade fever (temperature 37.8 C) whilst other vital signs were stable (blood pressure 134/92mmHg, heart rate 92 beats per minute, respiratory rate 22 breaths per minute, oxygen saturations 97%). Chest radiograph confirmed the presence of bilateral lower lobe pulmonary infiltrates (Figure 1). Blood investigations revealed a leukocytosis, neutrophilia, and an elevated C-reactive protein (CRP) (Table 1). Blood cultures were negative and arterial blood gases were normal. Additionally, a coronavirus disease 2019 (COVID-19) polymerase chain reaction (PCR) test was negative. A tentative diagnosis of persistent hiccups secondary to aspiration pneumonia was made. The patient was immediately started on an intravenous antibiotic regime consisting of amoxicillin/clavulanic acid 1g at 12-hour intervals and metronidazole 400mg dosed at eight-hour intervals. Additionally, the patient was placed on a low-dose chlorpromazine infusion (25mg chlorpromazine diluted in 1000ml 0.9% saline infused over four hours), and his blood pressure was closely monitored. 

**Table 1:** Laboratory results of the patient on admission and prior to discharge. HbA1C: hemoglobin A1C, pO2: partial pressure of oxygen, pCO2: partial pressure of carbon dioxide, HCO3: bicarbonate

Blood Parameters	Result on Admission	Result prior to Discharge	Reference Range
Haemoglobin (g/dL)	15.90	15.76	13.0-18.0 g/dL
White Blood Cells	14.06	8.79	4 – 11 x 10^9^/L
Neutrophil %	84.60	72.70	37 – 80%
Lymphocyte %	12.70	13.40	10 – 50%
Platelets	420	398	150 – 400 x 10^9^/L
Sodium	135	137	135 – 145 mmol/L
Potassium	4.7	4.3	3.5 – 5.3 mmol/L
Chloride	100.2	101.3	95 – 105 mmol/L
Urea	18.32	16.7	6.0 – 23.0 mmol/L
Creatinine	1.10	1.08	0.7 – 1.2 mmol/L
C-Reactive Protein	25.477	14.426	< 10 mg/L
Troponin	0.01	-	< 0.1mcg/L
HbA1C %	7.9	-	4.8 – 5.9%
pH	7.40	-	7.35 – 7.45
pO2	94	-	80-100 mmHg
pCO2	33.8	-	35 – 45 mmHg
HCO3	21.2	-	22 – 26 mmol/L

The patient experienced a near-complete resolution of his hiccups within 36 hours of treatment and a complete resolution prior to discharge four days later. Laboratory investigations were repeated prior to discharge and a decrease was noted in both CRP and leukocytes (Table 1). The patient was transitioned to a 10-day course of oral amoxicillin/clavulanic acid and metronidazole. At outpatient follow-up 14 days later, the patient is well and had no recurrence of the hiccups. 

## Discussion

Hiccups are defined as spasmodic, reflexive, and intermittent diaphragmatic and intercostal contractions that occur against a closed glottis [7]. It is typically benign, temporary, and self-limiting however if prolonged, it may represent an underlying pathology. Hiccups are labelled persistent if it occurs beyond 48 hours and intractable if it occurs continuously for more than one month [8]. Common causes of persistent hiccups include gastroesophageal reflux disease (GERD), empyema, diaphragmatic hernia, stroke (lateral medullary syndrome) and certain antipsychotic medications [9]. Lower lobe pneumonia can also cause persistent hiccups. Our case is interesting as hiccups were the patient’s sole symptom and typical symptoms of aspiration pneumonia were absent. 

This unique manifestation of pneumonia may be influenced by the position of the patient during the aspiration episode. The right lung is commonly affected in aspiration syndromes since the right main stem bronchus is wider, shorter, and more vertical than the left [10]. With patients in the upright position, aspiration of gastric contents commonly involves the basal segments of the right lower lobe [5].

The pathophysiology of persistent hiccups arising after aspiration may be explained by a two-step process following involvement of the basal lung lobes. According to Mendelson et al., in the first step, aspiration of acidic gastric contents combined with oropharyngeal or enteric micro-organisms produces a direct corrosive effect on the epithelial surfaces of the airway, particularly in the basal segments of the right lung [11,12]. Commonly implicated micro-organisms involved in aspiration syndromes include gram-negative bacilli and anaerobes such as Fusobacterium, Bacteroides and Peptostreptococcus [13]. The second step begins four to six hours later with a neutrophilic inflammatory reaction characterised by the attraction of inflammatory mediators, polymorphonuclear cells and the production of reactive oxygen species [11]. This interstitial and alveolar inflammatory reaction may directly irritate both the afferent and efferent limbs of the phrenic nerve which produces diaphragmatic spasms and persistent hiccups [9]. This may explain why our patient’s persistent hiccups began several hours after the episode of vomiting.

Patients with aspiration pneumonia typically present with dyspnea, cough, fever, hypoxemia, and radiological evidence of pulmonary infiltrates [14]. On clinical examination, decreased breath sounds or crackles are noted in the basal lung segments [3]. However, in the elderly, typical signs and symptoms of pneumonia may be absent. According to Makhnevich et al., the triad of cough, dyspnea and fever is present in only 31% of elderly patients [15]. When hiccups are the only symptom, laboratory investigations and chest radiography are important in establishing the etiology [16]. In our case, chest radiograph revealed infiltrates in both lower lung lobes and laboratory investigations displayed an evolving inflammatory process with an elevated C-reactive protein, leukocytosis, and neutrophilia. These findings, together with the onset of hiccups hours after post-prandial emesis led to the diagnosis of aspiration pneumonia.

The treatment of persistent hiccups is geared towards the underlying etiology and in our case, this included chlorpromazine and antibiotics. Chlorpromazine is currently the only medication approved for hiccups by the US Food and Drug Administration (FDA) [17]. Chlorpromazine is a phenothiazine derivative and antipsychotic, which acts as a central dopamine antagonist in the hypothalamus and produces combined blockade at the histamine H1, muscarinic M1 and dopamine D2 receptors in the vomiting centre. Hypotension is a serious adverse effect of this drug; therefore, treatment is initiated at a low dose and close blood pressure monitoring is required [17]. The treatment dose for persistent hiccups is 25-50mg orally, intramuscularly, or intravenously every four to six hours [18]. Empiric broad-spectrum antibiotic therapy is imperative in amelioration of the insult involving the hiccup reflex arc. A typical regimen for aspiration pneumonia includes penicillin combined with metronidazole for coverage against enteric anaerobic organisms [13].

## Conclusions

In conclusion, this case highlights an unusual presentation of aspiration pneumonia. Although hiccups are benign and self-limiting, it is important that clinicians understand that persistent and intractable hiccups represent a serious underlying pathology. In the elderly, typical signs and symptoms of aspiration pneumonia may be absent. When pneumonia involves the basal lung lobes, persistent hiccups may arise from diaphragmatic irritation, and treatment should be geared towards symptomatic relief and the inciting insult. 
